# The Future of Neurorehabilitation: Putting the Brain and Body Together Again

**DOI:** 10.3390/brainsci13121617

**Published:** 2023-11-22

**Authors:** Marco Iosa, Stefano Paolucci, Giovanni Morone

**Affiliations:** 1 Department of Psychology, Sapienza University of Rome, 00185 Rome, Italy; 2 Santa Lucia Foundation, Scientific Institute for Research, Hospitalization and Health Care (IRCCS), 00179 Rome, Italy; s.paolucci@hsantalucia.it; 3 Department of Life, Health and Environmental Sciences, University of L’Aquila, 67100 L’Aquila, Italy; giovanni.morone@univaq.it; 4 San Raffaele Institute of Sulmona, Viale dell’Agricoltura, 67039 Sulmona, Italy

The neurorehabilitation of cerebrovascular diseases is a challenging scientific topic that has rapidly grown in recent decades. For years, neuromotor physiotherapy aimed at improving functional recovery and cognitive/psychological therapy aimed at enhancing cognitive and behavioural functionality have been applied as distinct interventions aimed at addressing different functions. However, the direct impacts of cognitive and behavioural complications on the quality of life of patients, as well as the indirect influence of cognitive deficits on motor skills, deserve more attention from the scientific community [1,2].

A noteworthy effort is required in the field of neurorehabilitation in which therapy segmentation has been extensive due to cultural, historical, and organizational reasons. Currently, the advent of new technologies highlights the limitation of this division, which separates motor and cognitive recovery planning [3,4].

The sharp distinction between mind and body was already criticized in the late 1990s in some famous books, including those by Damasio (*Descartes’ Error*) [5], Clark (*Being There—Putting Brain, Body and World Together Again*) [6], and Berthoz (*Le Sens du Mouvement*) [7]. Nevertheless, neurorehabilitation seems to have been slow in incorporating these ideas into its protocols.

The aim of this Special Issue is to provide an overview of the role of cognition in neurorehabilitation after a cerebrovascular event, especially executive functions, personal unilateral neglect, and motor imagery, with particular attention paid to the possible role of new, emerging technologies.

In this Special Issue, Tarantino et al. [8] show how the combination of training focused on executive functions with an ordinary rehabilitation program potentiates beneficial effects in promoting independence in the activities of daily living. This independence may also benefit from the reduction in unilateral spatial neglect obtained through prism adaptation therapy [9]. An intervention that involves perception and action, such as Action Observation Therapy, can contribute to increasing motor recovery in subacute stroke patients with moderate-to-severe upper limb impairment in the early phase after stroke [10]. Bobrova and colleagues [11] report that the motor imagery of the hand, upon which some rehabilitation protocols are based, may depend on personality traits. All of these findings confirm the importance of cognitive recovery, which has been found to be related to serotonin levels, in patients with stroke [12]. In this scenario, it becomes also essential to assess the participation of patients with stroke in their own rehabilitation, and the article by Iosa and colleagues may contribute to the diffusion of the Pittsburgh Rehabilitation Participation Scale [13]. Another aspect that can be evaluated is oral health; in fact, poor oral health status was found to be associated with inpatient rehabilitation outcomes by Gerreth et al. [14].

Regarding the perception–action link, more studies are needed to investigate the potential effects of Vibrotactile-Based Rehabilitation on balance and gait recovery in patients with neurological diseases, as stated by De Angelis and colleagues in their systematic review [15]. Similarly, the review by Amoros-Aguilar et al. recommends conducting more rigorous studies to explore the potential benefits of combining aerobic exercise and cognitive training in the rehabilitation of patients with stroke [16].

A counterproof regarding the importance of cognitive functions in motor recovery is provided by the study conducted by Aprile and colleagues [17]. They demonstrate that deficits in spatial attention and executive functions reduce improvements in independence in the activities of daily living. Additionally, language, number processing, and spatial attention deficits hindered gains in the recovery of upper extremity functions in patients with stroke treated with upper-limb robotic rehabilitation. For lower-limb rehabilitation, the use of Overground Robot-Assisted Gait Training achieved the same level of efficacy as conventional rehabilitation [18]. Regarding new, emerging technologies, a special mention is deserved for artificial intelligence, which is discussed by Iosa and colleagues in a paper showing how a neural network can assist in identifying prognostic factors for stroke rehabilitation [19].

In light of the interesting papers published in this Special Issue, the future of neurorehabilitation appears to be linked to a multidisciplinary approach that transcends subfield divisions subfields and very specific techniques aimed at specific single functions, focusing instead on the patient as a whole person and putting the brain and body together again.
